# A new strategy for better genome assembly from very short reads

**DOI:** 10.1186/1471-2105-12-493

**Published:** 2011-12-30

**Authors:** Yan Ji, Yixiang Shi, Guohui Ding, Yixue Li

**Affiliations:** 1 Bioinformatics Center, Key Laboratory of Systems Biology, Shanghai Institutes for Biological Sciences, Chinese Academy of Sciences, Shanghai 200031, P.R. China; 2 Shanghai Center for Bioinformation Technology, Shanghai 200235, P.R. China

## Abstract

**Background:**

With the rapid development of the next generation sequencing (NGS) technology, large quantities of genome sequencing data have been generated. Because of repetitive regions of genomes and some other factors, assembly of very short reads is still a challenging issue.

**Results:**

A novel strategy for improving genome assembly from very short reads is proposed. It can increase accuracies of assemblies by integrating *de novo *contigs, and produce comparative contigs by allowing multiple references without limiting to genomes of closely related strains. Comparative contigs are used to scaffold *de novo *contigs. Using simulated and real datasets, it is shown that our strategy can effectively improve qualities of assemblies of isolated microbial genomes and metagenomes.

**Conclusions:**

With more and more reference genomes available, our strategy will be useful to improve qualities of genome assemblies from very short reads. Some scripts are provided to make our strategy applicable at http://code.google.com/p/cd-hybrid/.

## Background

In the past a few years, several new platforms, such as Roche 454, Illumina/Solexa and ABI SOLiD, which are called Next-Generation Sequencing (NGS) technology in general, have revolutionized the sequencing landscape. Compared to the traditional Sanger sequencing method, the NGS technologies have several distinct features. First, the lengths of NGS reads are shorter. A typical read from Sanger sequencing is about 650-800 base pairs. Roche's 454 sequencer produces reads between 250-400 bp, and Solexa/SOLiD reads are generally within 100 bp. Second, the NGS technologies enable one machine to simultaneously produce millions of reads. For example, the Roche/454's GS FLX Titanium, Illumina/the Solexa's GAII and Life/APG's SOLiD 3 can generate about 0.45, 4 and 7 Giga-bytes data in one run [1]. With the dramatically reduced time and cost for sequencing a genome, thousands of such projects have been finished or are in progress. These projects are either *de novo *sequencing or re-sequencing of prokaryotes and eukaryotic species (Genomes Online Database, http://www.genomesonline.org/). The NGS technologies were first applied to bacterial genomes [2-4]. For eukaryotic genomes sequenced through the NGS technologies, the giant panda genome was solely assembled from Solexa reads [5]; the filamentous fungus *Grosmannia clavigera *[6] and the cucumber *Cucumis sativus *[7] were sequenced in combination with the Sanger technology; and the genome of filamentous fungus *Sordaria macrospora *was assembled from a mixture of Solexa and 454 reads [8].

Genome assembly from very short reads is challenging because of genomic repeats and it also requires intensive computation resources. Two strategies are commonly used, the comparative assembly strategy and the *de novo *assembly strategy. For the comparative assembly strategy, DNA fragments are mapped to the reference and this information is used to infer the structure of genome being sequenced [9,10]. The *de novo *assembly strategy is to construct genome sequences from a set of sequence reads without the help of reference genomes, either using the overlap-layout-consensus (OLC) approach or an algorithm based on a de Bruijn graph (DBG). Both methods have been well described in previous reports [11,12]. Because the DBG-based assemblers can more accurately resolve genomic repeats with less computation than OLC-based ones, they have been widely adopted by genome sequencing projects [11].

The qualities of genome assemblies are evaluated by their contiguity and the accuracy of contigs or scaffolds [11]. The contiguity refers to lengths of contigs or scaffolds, such as the total length, the average length and the longest length, *etc*. The accuracy mainly means mis-assembly rates. Previous studies showed that, when the lengths of the NGS reads are shorter than genomic repeats, the complexity of genomic repeat regions is the major contributing factor to the quality of genome assembly [13-15]. Whiteford and colleagues showed that NGS reads of 30 bps could generate useful assemblies and recover almost all genes, while genes that failed to be correctly assembled are mostly related to repetitive elements (such as transposons, IS elements and prophages) [14]. Alkan and colleagues discovered that many genomic repeats or segmental duplications were left out by *de novo *assemblies of human genomes from short reads, and suggested to combine high-quality sequencing approaches with high-throughput ones for improving the assembly qualities [15].

There are several possible ways to improve the quality of a genome assembly from short read data. One is to utilize paired-end reads from libraries with different insert lengths [5]. Another is combining different types of reads such as Roche 454/Sanger and Solexa [6,8]. Using a reference genome to fill gaps between scaffolds of *de novo *assemblies may also be feasible [16,17]. The first two approaches work because either separation distances of paired reads or assemblies from longer reads increase the chance to resolve genomic repeats correctly. If a reference genome is highly similar to the target genome, a comparative assembly gets a better result than *de novo *approach because it is easier for it to resolve genomic repeats [10]. In some studies, comparative assemblies were also used to improve the quality of *de novo *assemblies [16,17]. As shown in the Result section, currently the comparative approach is limited by the availability of closely related reference genomes. If the similarity between the reference and the target genomes is not so high, as shown in the result section, contigs may be wrongly assembled.

Here, a novel strategy for improving the quality of genome assembly from very short reads is proposed. By combining *de novo *assemblies and comparative ones, this strategy can produce high quality assemblies in terms of both the contiguity and the accuracy. Among the major DBG-based assemblers, the ways they deal with genomic repeats and sequencing errors are different [18,19]. Therefore, their assembly results from short read data are different, as shown in the result section. Moreover it was discovered that mis-assembled contigs were still produced by Velvet [20], ABySS [21] or SOAPdenovo [22]. In our approach, a method is used to choose contigs from *de novo *assemblies, and these contigs are called DBG contigs. Using simulated short read datasets, we show that this method significantly reduce error rates of *de novo *assemblies and produce extremely reliable DBG contigs. Also, multiple comparative assemblies are produced by choosing multiple reference genomes without limiting to those highly similar ones. Then a method based on DBG contigs is proposed to eliminate almost all the mis-assembled contigs from the comparative assemblies. By doing so, the remaining comparative assemblies are reliable and can be used to improve the qualities of *de novo *assemblies. Tested on simulated and real short read datasets, we show this workflow is useful for improving the quality of assemblies from very short reads for isolate microbial genomes and metagenomes.

## Results

### Algorithm: the pipeline of our strategy

**Figure 1:**
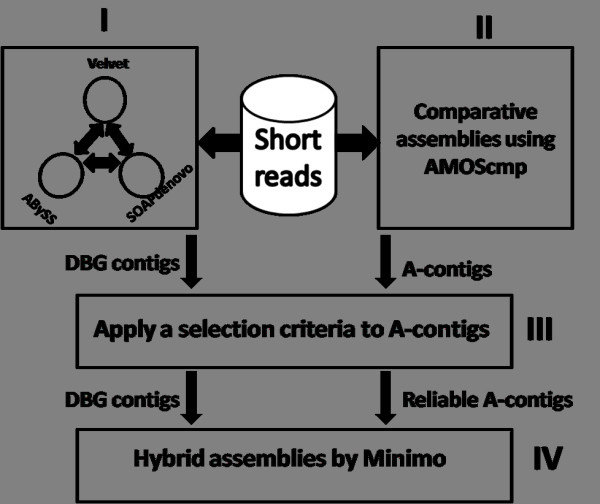
**The diagram of the pipeline of our novel strategy for genome assembly from short reads**.

Here, the pipeline of our strategy for assembling genomes from very short reads is described. As shown in the Figure 1, there are four modules in the pipeline. In the first one, short reads are processed by three DBG-based assemblers Velvet, ABySS and SOAPdenovo separately. From the three *de novo *assemblies, a contig is chosen only if it is identical to or a subsequence of contigs from at least two of the *de novo *assemblies. The resulting contigs are called DBG contigs. In the second module, multiple genomes are selected as references and short reads are assembled using the comparative assembler AMOScmp [10]. The contigs obtained through this step are called A-contigs. Because there are probably mis-assembled A-contigs, a method is devised to eliminate them in the third module. After DBG contigs are aligned onto A-contigs, an A-contig must meet two requirements to be considered reliable. First, there are no significant insertions or deletions in all its alignments. Second, it should be covered by enough DBG contigs so that the proportion of summed lengths covered by the DBG contigs should be over a threshold. In this paper, the threshold is set to 0.8. These contigs are called reliable A-contigs. In the last module, the mixture of DBG contigs and reliable A-contigs are assembled by Minimo [23,24].

### The quality of DBG contigs

**Figure 2:**
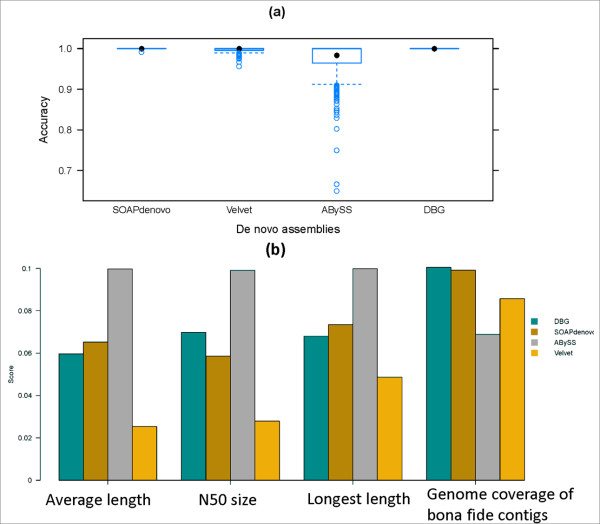
**Using simulated short read datasets from 629 genomes, comparisons among four types of *de novo *assemblies which get contigs longer than 500 bp**. (a) Box-and-whisker plots of accuracies for the four types of *de novo *assemblies. Accuracy is the proportion of *bona fide *contigs in a genome assembly (b) Comparisons among the four types of *de novo *assemblies in terms of the average length, the N50 size, the longest length and the proportion of genome length covered by bona fide contigs.

Using simulated short read datasets from 629 genomes, 629*4 *de novo *assemblies were produced by the first module of the pipeline for the Velvet assembly, the ABySS assembly, the SOAPdenovo assembly and the DBG assembly. In Figure 2a, the four types of assemblies are compared in terms of their accuracies which are defined as the proportion of bona fide contigs. Bona fide contigs can be easily identified by mapping assembly contigs to the genome for simulation. It is shown that 287 velvet assemblies, 458 ABySS assemblies and 1 SOAPdenovo assemblies contained at least one inaccurately assembled contigs. In contrast, our approach performs well to eliminate mis-assembled contigs since all those misassembled contigs from the Velvet and the ABySS assemblies are not chosen as DBG contigs. In Figure 2b the four types of assemblies are compared in terms of four criteria, i.e., the average length (the total length divided by the number of contigs), the N50 size (after sorting contigs according to their lengths in descending order, the length of the first contig such that the sum of contigs of equal length or longer is at least 50% of the length of a genome used for simulation), the longest length (the maximum lengths of contigs) and the proportion of genome length covered by bona fide contigs. The four criteria are computed from all contigs of the 629 simulated assemblies. For each simulation dataset from a genome, an assembly method or tool gets a rank of 1 to 4 in each category. Top ranked ones will have the highest weight (in our case, this weight is assigned as 4), and the bottom ranked ones will have the lowest weight (1 in our case). The ones ranked the second and the third will get their weights accordingly too. The score in Figure 2b (vertical axis) is the sum of the weight-adjusted the placements in the 629 tests (Σ the times ranked i * weight_i_, for i = 1 to 4) for each assembly method or tool. The ABySS assembly outperforms other three assemblies in all the categories except the proportion of genome length covered by bona fide contigs, and the DBG assembly has a similar performance to the SOAPdenovo assembly. But, Figure 2a shows the ABySS assembly has the worst performance in terms of the accuracy. Therefore, our method to select contigs from *de novo *assemblies not only results in the extremely accurate DBG assembly, but also gives a good assembly quality in terms of contiguity. In addition, this method makes it possible to extend our novel strategy if more DBG-based *de novo *assemblers emerge in the future.

### Choosing reference genomes

**Figure 3:**
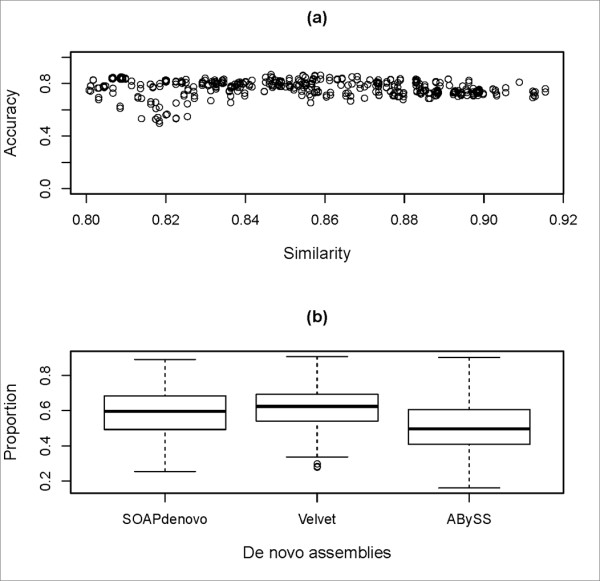
**Reasons for choosing reference genomes not highly similar to a target genome**. (a) Similarity is the similarity value between a reference and a target genome. Accuracy is the proportion of *bona fide *contigs in a comparative assembly. (b) Given 312 comparative assemblies, proportions of *de novo *assemblies extended by them are shown using Box-and-whisker plots.

Usually, a genome is chosen as the reference only if the similarity between it and the target genome is close to 100%. This restriction leads to quite limited application of the comparative assembly. In our strategy, multiple reference genomes are chosen, even though some of them are not highly similar to the target. Using the software AMOScmp to assemble simulated short read datasets, as shown in Figure 3a, when similarities between target and reference genomes are within 0.80~0.92, accuracies of 312 comparative assemblies range from 0.50 to 0.87, averaging 0.77 and the standard deviation is 0.062. If mis-assembled contigs are excluded, the remaining bona fide contigs from comparative assemblies can be used to improve the quality of *de novo *assemblies. Using the same simulated short read datasets, proportions of contigs from *de novo *assemblies (the SOAPdenovo assembly, the ABySS assembly and the Velvet assembly) extended by comparative assemblies are shown in the Figure 3b. Proportions of the SOAPdenovo contigs extended by comparative assemblies range from 0.26 to 0.90, averaging 0.59 and the standard deviation are 0.13. Proportions of the Velvet contigs extended by comparative assemblies range from 0.28 to 0.91, averaging 0.62 and the standard deviation are 0.11. Proportions of the ABySS contigs extended by comparative assemblies range from 0.16 to 0.90, averaging 0.51 and the standard deviation are 0.15. It shows that the SOAPdenovo and the Velvet assemblies are similar in this regard, and they are both significantly different from the ABySS assemblies. There is an explanation for this. Compared to the contigs from the SOAPdenovo or the Velvet assemblies, contigs from the ABySS assemblies are always longer so that it is less likely for them to be extended. Therefore, even if reference and target genomes are not highly similar, comparative assemblies can still be used to improve the qualities of *de novo *assemblies after the mis-assembled contigs are excluded.

### Algorithm: criteria for selecting A-contigs

**Figure 4:**
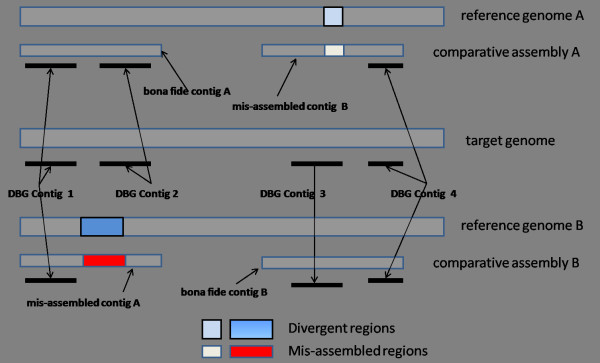
**It illustrates the criteria for selecting reliable A-contigs**. In the middle is the target genome which takes two genomes A and B as references. Because of divergent regions between the target and reference genomes, mis-assembled contigs A and B were produced. For mis-assembled contig A, a DBG contig fails to align to it; for mis-assembled contig B assembled against reference A, because the best mapped reference contig of DBG contig 3 is the bona fide contig B, its coverage of DBG contigs is smaller than the one assembled against reference B.

The criteria for selecting A-contigs are illustrated in Figure 4. Because of the distinct divergent regions on the reference genomes A and B, two mis-assembled contigs A and B and two bona fide contigs A and B are produced by the comparative assembly strategy. Four DBG contigs 1-4 are produced by the *de novo *assemblies. The mis-assembled contig A is excluded because it fails to align the DBG contig 2 onto it. Because the DBG contig 3 aligns with the bona fide contig B better than it does with the mis-assembled contig B, the mis-assembled contig B is excluded due to its lower coverage of the DBG contigs.

**Figure 5:**
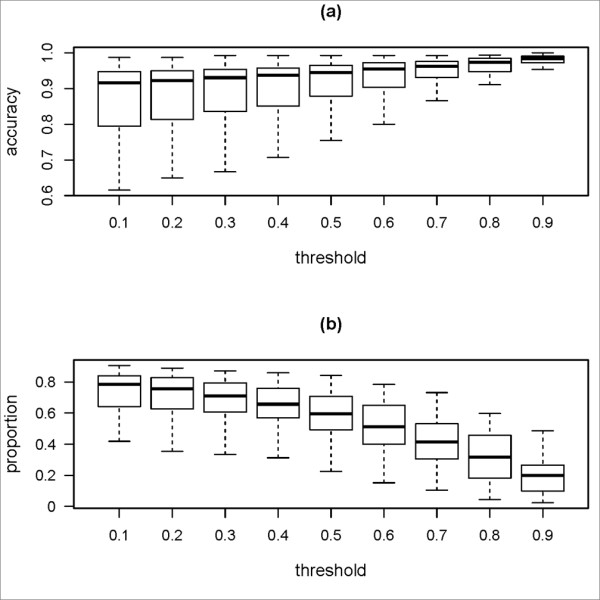
**The effect of the threshold used for comparative assemblies on the accuracy of reliable contigs**. Using simulated short read datasets from 41 genomes which take at least three genomes as references, comparative assemblies are produced by AMOScmp. (a) for different thresholds, different sets of reliable A-contigs are obtained and their accuracies are shown using the Box-and-whisker plots. (b) Correspondingly, proportions of the DBG contigs extended by reliable A-contigs are shown using the Box-and-whisker plots.

To exclude the mis-assembled contigs, we need to set a threshold for the coverage of DBG contigs. It is an important parameter since it affects the accuracies of chosen reliable A-contigs as well as the usefulness of these contigs to improve the quality of genome assembly. In Figure 5a, it is shown that, the higher the threshold from 0.1 to 0.9, the better accuracies of reliable A-contigs. For example, after excluding mis-assembled contigs by a threshold of 0.1, about fifty-nine percent of the reliable A-contigs have accuracies better than 0.90. When the threshold is set to 0.8, the number increases to about ninety percent. However, as shown in Figure 5b, the proportions of DBG contigs extended by reliable A-contigs will decrease when the threshold is higher. For example, at the threshold 0.8, the proportion of DBG contigs extended is significantly lower than that when the threshold is 0.1. In the following sections, we will demonstrate that qualities of genome assemblies are greatly improved even with a stringent threshold, when tested on simulated or real short read datasets.

### Testing: validation of our strategy

**Figure 6:**
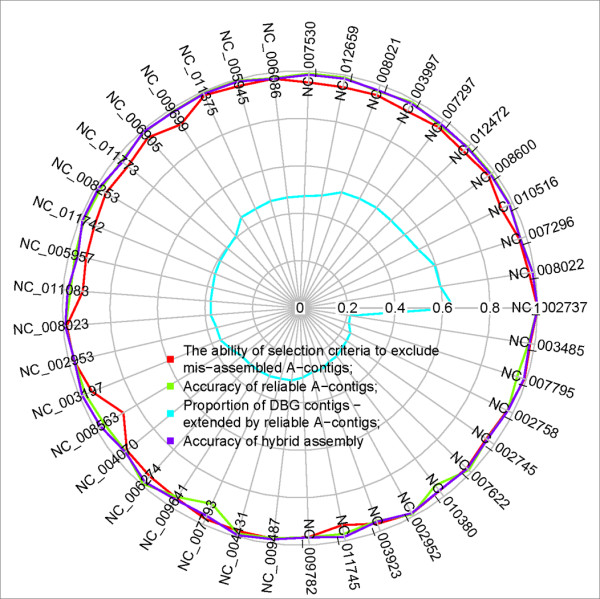
**Validation of our strategy using simulated short read datasets**. Simulated short read datasets from 41 genomes are used, and for each dataset we select at least three reference genomes. The similarity values between the reference and the target genomes are between 0.80 and 0.92. We compute the ability of the selection criteria to exclude misassembled A-contigs, the accuracy of reliable A-contigs, the accuracy of hybrid assembly and the proportion of DBG contigs extended by reliable A-contigs.

Essentially, our strategy provides a way to generate more comparative assemblies and use them to improve qualities of *de novo *assemblies. Two aspects should be validated, the accuracy and effectiveness of comparative assemblies. As shown in Figure 6, three measures are computed to evaluate the accuracy of comparative assemblies. First is the ability to exclude misassembled A-contigs, i.e., the ratio of the number of excluded mis-assembled A-contigs to the number of total mis-assembled A-contigs. Our selection criteria excludes more than 95% misassembled A-contigs for 91% simulated datasets. Second is the accuracy of reliable A-contigs, for nearly 100% of the simulated datasets they are bigger than 0.9. Third is the accuracy of hybrid assembly from a mixture of reliable contigs and DBG contigs, for nearly 100% of the simulated datasets they are higher than 0.95. Moreover, variations of the proportions of DBG contigs extended by reliable A-contigs of chosen genomes in the Figure 6 can be partly explained by genome complexity values and similarities of target genomes with their reference genomes. First, the Pearson correlation value between genome complexity values and such proportion was -0.4. So, low proportions are partly caused by genomes of big complexity. Second, given comparative assembly tool AMOScmp, the reference genome is another contributing factor to such variations. Because the Pearson correlation value between average similarities of target genomes with their reference genomes and such proportions is 0.4. So, bigger proportions are also partly due to bigger similarities of target genomes with their reference genomes.

**Figure 7:**
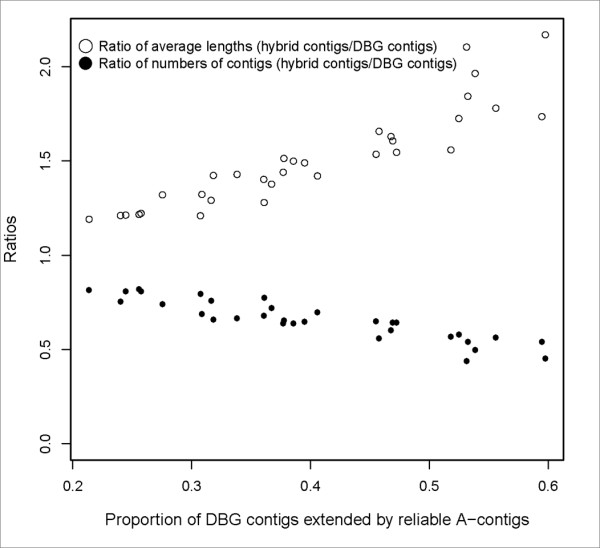
**Comparisons between hybrid assemblies and DBG contigs in terms of the average length and the number of contigs**. The simulated short read datasets are the same as the ones used for figure 6.

The effectiveness of comparative assemblies is measured by the proportion of *de novo *contigs which can be extended by comparative assemblies. When the threshold used to select reliable A-contigs is set to 0.8, such proportions are mainly between 0.2 and 0.6. After assembling reliable A-contigs and DBG contigs by Minimo, hybrid assemblies are produced and their contigs are compared with the DBG contigs. In Figure 7, two kinds of ratios are shown. One is the ratio of the number of contigs of hybrid assemblies to the number of DBG contigs. The other is the ratio of the average length of contigs of hybrid assemblies to the average length of DBG contigs. Compared to the DBG contigs, when the aforementioned proportions increase from 0.2 to 0.6, the number of contigs of hybrid assemblies drops while their average lengths increase. It demonstrates that our strategy can improve the quality of genome assembly on simulated short read datasets.

### Testing: application of our strategy

#### Isolate microbial genome assembly

After filtering out the low-quality reads, our pipeline is used to assemble paired-end reads randomly sampled from short reads of *Bacillus subtilis subsp. natto BEST195 *(SRA: DRX000001) [25]. A draft assembly (Nucleotide: AP011541) of strain *Bacillus subtilis subsp. natto BEST195 *(Taxonomy: 645657) from very short reads (36 bp) was produced by combining sequences from both the Velvet assembler and the MAQ software.

In the first module, DBG contigs are produced from separated Velvet, ABySS and SOAPdenovo assemblies. In the second module, three genomes are chosen as references, *Bacillus subtilis subsp. subtilis str. 168 *(Nucleotide: NC_000964; Taxonomy: 224308), *Bacillus subtilis subsp. spizizenii str. W23 *(Nucleotide: NC_014479; Taxonomy: 655816) and *Bacillus subtilis BSn5 *(Nucleotide: NC_014976; Taxonomy: 936156). Their blast coverages against AP011541 are 86%, 83% and 87%, respectively. Short reads are assembled by AMOScmp against reference genomes and give A-contigs. In the third module, reliable contigs are chosen. Finally, a hybrid assembly is produced through the fourth module.

In the table 1, the total length of the DBG contigs is 3,927,655, and they cover 96.34% of nucleotides of *B. subtilis natto*'s draft assembly. Among the 207 DBG contigs longer than 1000 bp, four fill 63,804 gaps in the scaffolds of *B. subtilis natto*'s draft assembly. The total length of the reliable contigs is 3,754,796, and there are 1068 contigs longer than 500 bp. After aligning reliable A-contigs onto DBG contigs, it is estimated that at least 100 DBG contigs are extended or merged by A-contigs. For the hybrid assembly, both the total length and the average length increase compared to the DBG contigs or the reliable A-contigs. The number of contigs drops to 94, and 282,652 gaps in the scaffolds of *B. subtilis natto*'s draft assembly are filled.

**Table 1:** Results when our novel strategy is applied to a real short read dataset

Measurements Assemblies	Total length	Average length	Contig number (> 1 kbp)	Longest length
**Velvet**	3,912,568	15,840	247	162,643
**ABySS**	4,099,096	35,035	117	243,520
**SOAPdenovo**	3,919,811	13,851	283	154,821
**DBG**	3,927,655	18,974	207	241,821
**Reliable A-contigs**	3,754,796	3,516	1068(> 0.5 kbp)	39,380
**Hybrid assembly**	4,304,581	45,793	94	241,821

#### Metagenome assembly

Metagenomics provides opportunities for in-depth investigating environmental microbes by directly sequencing their DNA materials randomly sampled [26]. Obviously the good quality of metagenome assembly will be helpful for metagenome researches, because longer sequences not only make gene prediction more accurate but also contain more genome context information to assist gene annotations. So far metagenome assemblies are still challenging, and most available *de novo *assemblers for reads of NGS techniques have a limited capability to assemble metagenomes [27]. The quality of *de novo *metagenome assembly is affected not only by repeats of the same or different genomes but also heterogenous DNA fragments of different coverages. The comparative assembly strategy is promising to improve the quality of metagenome assembly, but reference genomes of nearly 100% genome similarity with microbial members of metagenomes are hard to find since even genomes of the same species may not be the same, for example, genomes of various Escherichia coli species. Therefore, by allowing less similar genomes as references and thus choosing more references, our strategy makes it possible to assemble metagenomes in a comparative way.

In order to quantitatively show the ability of our strategy to improve metagenome assembly, two sets of simulated metagenomes of different overall coverages are tested. Five genomes of Escherichia coli species (NC_011745, NC_009800, NC_008253, NC_011415 and NC_009801) which are dominant in the human gut microbial communities are used to simulate metagenomes using Metasim [28] by equally sampling reads from each genome, and six genomes of the same species (NC_004431, NC_008563, NC_011741, NC_011742, NC_011748 and NC_012759) are chosen as references (genome similarity values between the five genomes and their references are listed in the additional file 1). In table 2, results of metagenome assemblies by our strategy and comparisons with other assemblers are shown. First, from the column "Mis-assembled number", our strategy significantly reduces the number of mis-assemblies caused by small sequencing coverages. Second, through comparisons between the row "DBG" which is the result of integrated de novo assemblies and the row "hybrid" which is the result of hybrid assembly of DGB contigs and reliable A-contigs, our strategy improves the quality of assemblies by increasing the total length, the longest length, the average length and reducing the number of contigs.

**Table 2:** Results when our novel strategy is applied to two sets of simulated metagenomes

	Total length		Longest length		Average length		Contig number		Mis-assembled number	
	**16×**	**32×**	**16×**	**32×**	**16×**	**32×**	**16×**	**32×**	**16×**	**32×**

**SOAPdenovo**	1,053,950	2,152,668	11,585	18,572	1,692	2,613	623	824	0	0
**Velvet**	3,541,492	3,479,492	17,858	13,775	2,359	2,359	1501	1475	9	6
**ABySS**	5,696,753	5,567,101	24,687	39,688	2,079	2,784	2740	2000	22	10
**DBG**	1,966,330	2,998,013	12,098	18,572	1,293	1,875	1520	1599	0	0
**Hybrid**	2,091,714	3,081,039	14,651	18,572	1,907	2,434	1097	1266	0	0

## Discussions

In our strategy, two key approaches are devised to improve the qualities of genome assemblies. In the first module, long contigs are selected from three *de novo *assemblies so that the error rates are largely reduced. This is based on the fact that the DBG-based assemblers adopt different approaches to resolve ambiguities in de Bruijn graphs caused by genomic repeats or other, so there are significant inconsistencies among sets of long mis-assembled contigs by different DBG-based assemblers. Using simulated short read datasets, this assumption is shown to be true for at least three assemblers (Velvet, SOAPdenovo and ABySS), since almost all mis-assembled contigs which are at least 500 bps in length are excluded by this method. Thus, this method can improve the accuracy of genome assembly. In the second and third modules, another approach is proposed to improve the quality of genome assembly in terms of their contiguity. It applies comparative assembly strategy in a broaden way, allowing multiple references without limiting to genomes of closely related strains. Most of the mis-assembled contigs generated through this step are then eliminated by the criteria used for selecting reliable comparative contigs. Tested on simulated and real short read datasets, we demonstrate that comparative contigs can indeed be used to extend or scaffold *de novo *contigs. Moreover, in this paper, accuracies of genome assemblies of different steps in the process of our novel genome assembly strategy have been graphically shown in Figure 2, Figure 3 and Figure 6. Genomes of either Figure 3 or Figure 6 were subsets of genomes of Figure 2. First, Figure 2a showed that accuracies of DBG contigs were 100% for all simulation datasets while there were wrongly assembled contigs in assemblies of Velvet, ABySS and SOAPdenovo. Second, as shown in Figure 3a and Figure 6, our criteria for selecting A-contigs significantly improved accuracies of A-contigs from average 77% to average 90%. Third, after assembling DBG contigs and reliable A-contigs, accuracies of hybrid assemblies were average 95%. Meanwhile the contiguity quality of genome assembly was significantly improved in comparison with de novo assemblies, as shown in Figure 7.

In practice, if other DBG-based assemblers are available, the method used to produce DBG contigs in the first module makes it possible to integrate results of more than three *de novo *assemblies. Moreover, at least two reference genomes should be chosen to produce comparative assemblies, because the criteria for selecting reliable A-contigs are specially designed for multiple reference genomes, and are expected to have a better performance with more comparative assemblies. In the fourth module, only a stringent light-weight assembler Minimo is used to assemble the mixture of DBG contigs and reliable A-contigs. Additional processing steps may be needed such as scaffolding using Bambus [29] and gap filling of the scaffolds using IMAGE [30].

For a genome sequencing project, if without genomes of closely related species and the *de novo *assemblies by DBG-based assemblers are highly fragmented, our strategy should be the first assembly pipeline to be tried. The effectiveness of our strategy depends on certain factors, for example, the complexity of repetitive regions of genome being sequenced and the similarity values between it and the chosen reference genomes. So, for some short read dataset, our strategy may not work, and other strategies are then considered.

In the future, we will try to integrate our strategy for selecting reliable comparative contigs and other signatures for assembly validation such as mate-pair orientations and separations and depth-of-coverage. We hope that, by eliminating almost all mis-assembled comparative contigs, more reliable A-contigs will be chosen to extend more DBG contigs so that qualities of genome assemblies can be further improved.

## Conclusions

A novel strategy for improving genome assembly from very short reads is proposed. The basic idea is that comparative assemblies can be used to improve qualities of genome assemblies by scaffolding or extending *de novo *contigs. *De novo *contigs are produced by integrating assemblies got by different DBG-based assemblers. Compared to assemblies by single assembler, error rates are largely reduced on simulated datasets. Comparative assemblies are produced by allowing multiple references, not limiting to closely related genomes. A method is proposed to exclude mis-assembled contigs generated due to this reduced similarities between reference and target genomes.

With more and more microbial genomes available, our strategy will be useful to improve qualities of genome assemblies from very short reads. Some scripts are provided to make our strategy applicable at http://code.google.com/p/cd-hybrid/.

## Methods

### Codes for the pipeline

In order to make our strategy applicable, codes for the pipeline are provided and available from http://code.google.com/p/cd-hybrid/. The *generateDBGcontigs.pl *script can take *de novo *assemblies from different tools as inputs and gives out DBG contigs. The *chooseReliableAcontigs.pl *script can take DBG contigs and a set of comparative assemblies as inputs and produce reliable A-contigs.

### The method to choose genomes used for simulated short read datasets

A simple measure is used to estimate the complexity of genomic repeat regions. For a genome, a value is calculated by dividing the sum of lengths of all genomic repeats by the genome length. Some scripts in MUMmer software are used to identify repeats. Scripts and their parameters are "nucmer --maxmatch -nosimplify" and "show-coords -r -T -H".

For short read datasets used to show the quality of DBG contigs, 629 genomes of complexity values bigger than 6e-3 and lengths longer than 1e6 are chosen (see additional file 2). For short read datasets used for comparative assemblies, 41 genomes are chosen as targets using following criteria: their complexity values are bigger than 6e-3; their genome lengths are longer than 1e6; at least three reference genomes are available; similarity values for their reference genomes are larger than 0.8 (see additional file 3). The similarity value between target and reference genomes are calculated from sequence alignment results using 'mummer' script in MUMmer software. The similarity value is defined as the ratio of the sum of lengths of maximal matches between two genomes and the total length of two genomes. Comparative assemblies used to show the performance of the criteria for selecting A-contigs are produced by 41 target genomes with each of their reference genomes. Comparative assemblies used for validation of our strategy on simulated short read datasets are produced by 41 target genomes with all of their reference genomes.

### Simulated and real short read datasets

Given a genome of length G, a coverage C and insert length L, short reads of length R of forward-reverse paired-end libraries are simulated by sampling G*C/(2*R) stretches of sequences of length L start positions of which are uniformly distributed on the sequence of genome and then taking two sequences of length R from ends of stretches. In this paper, the C is 60, L is 300 and R is 75. The simulation processes are launched by Maq's simulation module [25].

For short reads of *Bacillus subtilis subsp. natto BEST195 *(SRA: DRX000001) the number of reads are 27,296,731 (982.7Mbp). After filtering out 488,869 reads quality scores of which containing characters 'N', 11,214,956 (403.7Mbp) reads randomly sampled from remaining reads are used for genome assembly.

### Running DBG-based assemblers, AMOScmp and Minimo

For the Velvet assemblies from simulated datasets, parameters are "velveth 29 -fastq -shortPaired" "velvetg -cov_cutoff auto -exp_cov auto -scaffolding yes". For the SOAPdenovo assemblies from simulated datasets, parameters are "SOAPdenovo-31mer all -K 29" and "reverse_seq = 0; asm_flags = 3; rank = 1; pair_num_cutoff = 3". For the ABySS assemblies from simulated datasets, parameters are "abyss-pe k = 29 n = 10" and "ABYSS -k 29". For all the *de novo *assemblies from the real short dataset, the value of kmer is replaced with 23.

For the comparative assemblies from both simulated and real datasets, the AMOScmp-shortReads tool is used.

For the hybrid assemblies from DBG contigs and reliable A-contigs by Minimo, parameters are "-D FASTA_EXP = 1 -D MIN_LEN = 30".

### Aligning sequences using MUMmer

Three scripts in MUMmer software are used to align sequences, nucmer, delta-filter and show-coords. Their parameters are "nucmer --maxgap = 500 --mincluster = 100 --maxmatch", "delta-filter -q" and "show-coords -T -c -l -o -r -H -I = 0.2". In this paper, some methods adopt this approach to align sequences, such as the one to remove redundant contigs from DBG contigs, the one to select reliable A-contigs from comparative assemblies by aligning DBGs and the one to identify mis-assembled contigs from *de novo *assemblies and comparative assemblies by aligning them onto genomes used for simulation. Annotations of alignments given by "show-coords" script are used to implement these methods, such as "[CONTAINS]", "[CONTAINED]" and "[IDENTITY]".

## List of Abbreviations

NGS: the next generation sequencing technology; DBG: De Bruijn graph.

## Authors' contributions

YJ designed the study, performed bioinformatics analyses. YS participated in data analyses and interpretation. YJ and YS wrote the manuscript. GD gave some suggestions. YXL supervised the study. All authors read and approved the manuscript.

## Supplementary Material

Additional file 1 **Metadata for metagenome assembly**. Additional file 1 lists genome similarity values between the five genomes and their references mentioned in the section Metagenome assembly. Click here for file

Additional file 2 **Genome data for Figure 2**. Additional file 2 lists 629 genomes which are used in the illustration of the quality of DBG contigs, shown in the Figure 2. Click here for file

Additional file 3 **Genome data for Figure 3, Figure 5, Figure 6 and Figure 7**. Additional file 3 lists 41 genomes which each has at least three reference genomes and is used for Figure 3, Figure 5, Figure 6 and Figure 7. Click here for file
